# Trained Immunity and HIV Infection

**DOI:** 10.3389/fimmu.2022.903884

**Published:** 2022-07-08

**Authors:** Dmitri Sviridov, Yury I. Miller, Michael I. Bukrinsky

**Affiliations:** ^1^ Laboratory of Lipoproteins and Atherosclerosis, Baker Heart and Diabetes Institute, Melbourne, VIC, Australia; ^2^ Department of Biochemistry and Molecular Biology, Monash University, Clayton, VIC, Australia; ^3^ Department of Medicine, University of California, San Diego, San Diego, CA, United States; ^4^ Department of Microbiology, Immunology and Tropical Medicine, School of Medicine and Health Sciences, The George Washington University, Washington, DC, United States

**Keywords:** HIV-1, Nef, exosomes, trained immunity, co-morbidities, inflammation, lipid rafts

## Abstract

Findings that certain infections induce immunity not only against the causing agent, but also against an unrelated pathogen have intrigued investigators for many years. Recently, underlying mechanisms of this phenomenon have started to come to light. It was found that the key cells responsible for heterologous protection are innate immune cells such as natural killer cells (NKs), dendritic cells, and monocytes/macrophages. These cells are ‘primed’ by initial infection, allowing them to provide enhanced response to subsequent infection by the same or unrelated agent. This phenomenon of innate immune memory was termed ‘trained immunity’. The proposed mechanism for trained immunity involves activation by the first stimulus of metabolic pathways that lead to epigenetic changes, which maintain the cell in a “trained” state, allowing enhanced responses to a subsequent stimulus. Innate immune memory can lead either to enhanced responses or to suppression of subsequent responses (‘tolerance’), depending on the strength and length of the initial stimulation of the immune cells. In the context of HIV infection, innate memory induced by infection is not well understood. In this Hypothesis and Theory article, we discuss evidence for HIV-induced trained immunity in human monocytes, its possible mechanisms, and implications for HIV-associated co-morbidities.

## Introduction

Trained immunity is an exciting new concept postulating that innate immune cells can acquire memory to a viral, bacterial or fungal infectious agent or other stimuli, such as toll-like receptor (TLR) agonists, oxidized LDL, and hormones, like aldosterone (1–5). This memory can influence innate responses to subsequent infection or stimulation by the same or even a different agent. The broad nature of trained immunity responses, similar to other functions of the innate immunity, is strikingly different from classical highly specific immune memory of T and B cells. Mechanistically, the innate memory is achieved by epigenetic and metabolic reprogramming that can be induced by a variety of agents *via* stimulation of pattern recognition receptors (1, 4, 6–12). Two alternative programs of trained immunity can be induced: training resulting in enhanced responses to second stimulation, and training resulting in tolerance, where the second response is suppressed. Which program would be engaged depends, at least in part, on the strength and length of the initial stimulation, as well as on the properties of the stimulating agent (13). The enhancing training program likely has evolved as an ancient mechanism to protect against pathogens, including viruses (14), whereas tolerance training program has evolved to prevent overactivation of innate immune responses upon sustained TLR stimulation (15). Although training originated as a defense mechanism, under certain circumstances, in particular when increased responsiveness of innate immunity cells is maintained for extended periods of time, it can have adverse consequences, as described for atherosclerosis, Alzheimer’s, and other chronic inflammatory diseases (5). In this article, we discuss the possibility of trained immunity initiated by HIV infection and ideas about its mechanisms and implications.

## Mechanisms of trained immunity

Mechanisms of training have been characterized by Netea’s group for β-glucan, a yeast cell wall constituent (16). Exposure of human monocytes to β-glucan, a TLR2 and TLR4 ligand, during the first 24 h of *ex vivo* differentiation, re-programs the cells in such a way that after a 6 day differentiation period into macrophages they exhibit a hypersensitivity to a number of TLR ligands. Moreover, by using genome-wide profiling of histone and DNA modifications (H3K4me3, H3K4me1, H3K27ac, and DNase I accessibility), the authors found that induction of the memory program was associated with a long-term epigenetic reprogramming of loci of the genes previously shown to be involved in activation of the innate immune system, including TNFα and IL-6 (17). In the case of β-glucan, the initiating event for epigenetic remodeling was found to be activation of the Akt-mTOR pathway, which upregulated glycolysis (18). One of the intermediate products of glycolysis, fumarate, is a negative regulator of the KDM5 histone demethylase, inhibition of which promotes trimethylation of the H3 histone on the K4 residue (H3K4me3). Another product of glycolysis, citrate, stimulates synthesis of Acetyl-CoA, which is a positive regulator of histone acetyltransferase and promotes acetylation of the H3 histone on the K27 residue (H3K27ac). Both modifications are associated with transcriptionally active chromatin. Acetyl-CoA also stimulates cholesterol biosynthesis, one of the intermediate products of which, mevalonate, is secreted and potently amplifies training by interacting with the insulin-like growth factor 1 receptor (IGF1R) that further stimulates the Akt pathway (18).

It remains unknown what features of an infectious agent are essential for inducing innate memory. It may well be that it is a general response to any infection, and the difference in reaction to different stimuli is only in the duration of the memory phenotype. Widespread protection against multiple infections characteristic to trained immunity was associated with several live virus-based vaccines, such as BCG or oral polio, and lasted from months to years (19, 20). This is consistent with an idea that time-limited exposure to the viral components induces not only a specific adaptive immune response, but also stimulates a much broader training of innate immune cells. The effect of chronic viral infection on innate immunity is unknown, but persistent inflammation associated with chronic HCV (21) or HIV (22) infection is consistent with an enhancing training rather than tolerance, and can be explained by an enhanced response of trained innate immune cells to TLR stimulation. Increased inflammatory responses may underlie pathogenesis of HIV-associated co-morbidities, making characterization of trained immunity during HIV infection a priority.

## Trained immunity in HIV infection

In the context of HIV infection, very few targeted studies of trained immunity have been performed so far. A recent study demonstrated that a live attenuated SHIV (a recombinant SIV carrying HIV envelope) induced protection against intrarectal challenge with pathogenic SIV in the absence of anti-envelope antibodies and independent of CD8 T cells, through a mechanism consistent with trained innate immunity of monocytes (23). Another study reported epigenetic modifications associated with pro-inflammatory phenotype and reduced anti-tuberculosis activity of monocytes from HIV-infected subjects, both ART-treated and untreated (24), supporting the idea of trained immunity. The viral protein initiating the training and the mechanistic details leading to epigenetic changes in myeloid cells were not investigated in these studies. A study by van der Heijden et al. reported overexpression of IL-1β by monocytes from HIV-infected individuals, which was sustained for at least a year, consistent with the trained immunity phenotype. The authors proposed circulating β-glucan as a causative agent (25). However, mechanistic conclusions in this paper relied on association studies, and lacked analysis of circulating HIV proteins.

As discussed above, the known mechanisms of trained immunity depend on activation of the Akt-mTOR pathway, which upregulates aerobic glycolysis and cholesterol biosynthesis (18). Increased glucose metabolism has been shown for monocytes from HIV-infected individuals, including ART-treated with undetectable HIV load (26). Among HIV-1 proteins, Nef is known to stimulate cholesterol biosynthesis (27), and has been established as the major pathogenic factor of HIV-1, responsible for many pathological features associated with HIV and SIV infection (28–33). Moreover, Nef, both endogenously expressed and exogenously added, has been shown to activate the Akt-mTOR pathway (34–37). We therefore hypothesize that Nef may initiate immune training in HIV-infected individuals. We propose that exposure of monocytes to extracellular Nef, either free or incorporated into extracellular vesicles (exNef), significantly increases production of inflammatory cytokines by monocyte-derived macrophages differentiated from these monocytes. This hypothesis implies that exNef induces immune training in monocytes, likely *via* the effects on target cell signaling pathways (35, 38, 39). Although the life span of circulating monocytes is relatively short (less than a week), upon migration into the tissues these cells acquire the phenotype of resident tissue macrophages with life span in months (40, 41). In addition, training of myeloid progenitor cells in the bone marrow would lead to a long-term production of trained monocytes, so if the same effect of exNef is demonstrated for bone marrow progenitor cells, it would provide a mechanism for persistent presence of trained monocytes in HIV-infected individuals. Access of exosomes to bone marrow progenitor cells *via* blood has been shown for melanoma-derived exosomes (42).

**Figure 1:**
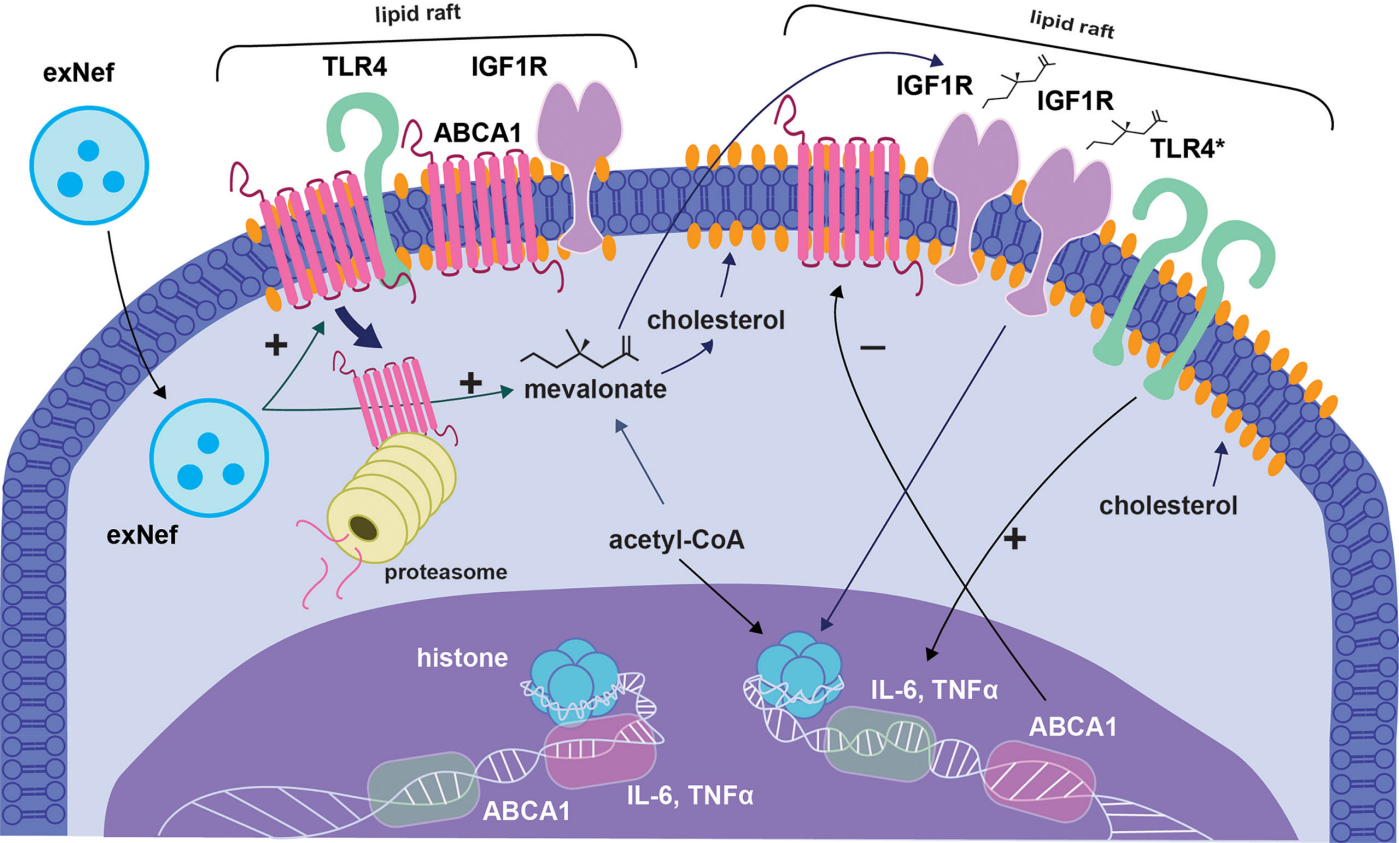
Proposed model of trained immunity induced by exNef. The figure shows suggested sequence of events occurring when monocyte encounters Nef-containing extracellular vesicle (exNef). ExNef induces proteasomal degradation of plasma membrane ABCA1, modifying lipid rafts and increasing their abundance. Lipid raft changes promote dimerization and activation of TLR4 (TLR4* in the figure), which sends activating signal to inflammatory genes. Additionally, exNef stimulates glycolysis and cholesterol biosynthesis. One of the intermediate products of glycolysis, fumarate, is a negative regulator of the KDM5 histone demethylase, inhibition of which promotes trimethylation of the H3 histone on the K4 residue (H3K4me3). Another product of glycolysis, citrate, stimulates synthesis of Acetyl-CoA, which is a positive regulator of histone acetyltransferase and promotes acetylation of the H3 histone on the K27 residue (H3K27ac). Both modifications are associated with transcriptionally active chromatin. These events lead to epigenetic modifications resulting in sustained changes in lipid rafts and inflammatory cytokine production. ExNef also suppress ABCA1 maturation and delivery to plasma membrane, resulting in decreased ABCA1 and suppressed cholesterol efflux. The cell becomes enriched in cholesterol, and the abundance of lipid rafts further increases, exacerbating events described above. An intermediate product of cholesterol biosynthesis, mevalonate, is secreted from the cell and stimulates the IGF1R, whose representation is increased on overabundant lipid rafts. Signaling from IGF1R stimulates the Akt-mTOR pathway, which further potentiates epigenetic remodeling. Histone modifications alter expression of lipid raft regulating proteins (ABCA1 is shown as an example, switching from active to suppressed state) and pro-inflammatory cytokines (IL-6 and TNF switch from suppressed to active state). Epigenetic changes in lipid raft regulating genes ensure persistent ‘activation’ of the lipid rafts, whereas epigenetic modifications of cytokine genes lead to overreaction to inflammatory stimuli. Together, these two mechanisms synergize to promote inflammation.

Interaction of exNef with macrophages impairs cholesterol efflux and elevates abundance of lipid rafts, promoting inflammatory responses (43, 44). Therefore, over responsiveness of exNef-treated monocytes mentioned above could be caused by a classical trained phenotype, i.e. epigenetic changes promoting expression of inflammatory genes, but also by sustained changes in the lipid rafts (**Figure 1**). Lipid rafts are considered highly dynamic and transient (45, 46). Upon cell activation, lipid rafts cluster and become more stable to accommodate agonist-induced receptors' assembly, such as homodimerization of TLR4, and initiation of signaling (47). These changes are usually transient. For example, in LPS-stimulated macrophages, TLR4 dimer-hosting lipid rafts last for 15 min and then disappear due to internalization of the LPS-TLR4 complex (48). A surprising discovery was that TLR4 dimers and increased lipid raft levels were found in spinal microglia for as long as 21 days after a chemotherapeutic intervention in mice (49). These results showing a long-term if not permanent maintenance of TLR4 dimers in microglia and macrophages under conditions of chronic neuroinflammation suggest that lipid rafts in these cells undergo reprogramming to condition for a fast and disproportionately strong inflammatory response. This may represent another form of trained immunity, which may be accomplished by epigenetic reprogramming of cholesterol metabolism genes involved in regulation of lipid rafts, rather than pro-inflammatory genes. Of course, these two possible mechanisms do not exclude each other.

Other HIV proteins may also contribute to HIV-induced trained immunity. Gp120 has been shown to activate signaling from TLR2 and TLR4 (50), suggesting that it may mimic the effects of β-glucan. Moreover, HIV-1 structural proteins p17, p24 and gp41 were found to function as pathogen-associated molecular patterns (PAMPs) for cellular TLR2 heterodimers (51). The HIV Tat protein activates a number of cellular signaling pathways, including the MyD88 and TRIF pathways originating from TLR4 (52). Out of these potential inducers of trained immunity, besides exNef, gp120 is the most likely candidate, as, in contrast to other mentioned proteins and similar to exNef, it can engage TLR receptors on an uninfected cell. Indeed, trained immunity in an infected myeloid cell is unlikely to have much of biological or pathological impact, simply because the number of such cells is very small (53). While gp120-induced trained immunity represents an interesting topic for investigation, we believe that it is unlikely to play a role in HIV co-morbidities as gp120 does not circulate in blood of ART-treated HIV-infected individuals.

## Trained immunity and HIV co-morbidities

Current antiretroviral therapy (ART) efficiently stops HIV replication eliminating viral particles from the blood and abolishing primary effects of the infection (e.g., immunodeficiency). Paradoxically, multiple co-morbidities of HIV infection, from neurological to cardiovascular and metabolic, persist (54). Risk of these co-morbidities in the HIV-infected population, although reduced compared to untreated HIV infection, is nonetheless increased 2-5-fold over the general population, making them the predominant cause of morbidity and mortality in people living with HIV. How a virus with a limited genome exerts such a profound and incessant effect on so many organs and systems, especially when the virus is not there? We believe that two explanations, which are not mutually exclusive, can be proposed.

While production of HIV particles is mostly supressed by ART, anti-retroviral drugs do not affect viral transcription and translation, allowing continued low-level expression of viral genes in the infected cells (55). In addition, viral proteins can be expressed from integrated ‘defective’ genomes, which represent majority of integrated HIV copies (56). These proteins are secreted into the bloodstream and surrounding tissues mainly in extracellular vesicles (EVs) (38, 57, 58). Nef may be the only or the major such protein produced in ART-suppressed individuals, as only Nef-specific T cells could be detected in people with HIV replication suppressed for several years (59). A recent study demonstrated that Nef at the concentrations of 5-10 ng/ml can be detected in blood of at least half of HIV-infected individuals with undetectable viral load (60). Using exNef at similar Nef concentration, we have recently showed that these vesicles are taken up by macrophages and neurons, modify their cholesterol metabolism, elevate abundance of lipid rafts and activate a number of inflammatory pathways (44, 61). This mechanism, together with slow recovery of the damaged mucosal tissue in the gut associated with leakage of bacterial products into the blood, may explain persistent inflammatory response (62). Leakage of bacterial products and LPS may also contribute to trained immunity (see below).

In the cited above study (44), we also tested if the effect of exNef on cholesterol metabolism disappears when Nef was removed after initial exposure. Contrary to our expectations, we found that the effects 48 h after removal of exNef were even stronger than in the presence of Nef. Further studies demonstrated that the effects of exNef on cholesterol metabolism and inflammation last for several weeks, far too long to be explained by residual Nef. Another explanation to this finding is that Nef triggers trained immunity, which is the second possible mechanism of persistent inflammation, consistent with findings discussed in the previous section. A recent paper invoked trained immunity to explain overexpression of IL-1β by monocytes from HIV-infected individuals, which was sustained for at least a year (25). In fact, this finding is consistent with both explanations discussed above, though Nef expression and epigenetic modifications were not tested in the study (25).

Most likely, the two mechanisms described above work together to establish the persistent inflammation observed in HIV-infected individuals. Indeed, hyperreactivity of myeloid cells due to exNef-induced changes in lipid rafts and trained immunity needs additional stimulation to accomplish the inflammatory response. This stimulation can be provided by bacterial and fungal products migrating from the gut.

Inflammation is an essential pathogenic element of many co-morbidities of HIV infection, and potentiation of inflammation by trained immunity should elevate the risk of these co-morbidities. Therefore, trained immunity in this case seems to play a pathogenic, rather than its usual protective role. The reasons for these unexpected and unorthodox properties of trained immunity in HIV infection deserve careful analysis, but it is clear that unique features of HIV infection, including rapid initial replication of the virus followed by low-level infection and latency, play the key role. The concept of trained immunity is not necessarily limited to inflammation. As discussed in the previous section, broader application of this concept, e.g. by including re-programming of lipid metabolism, allows to hypothesize that epigenetic modifications of genes for ABC transporters or sphingomyelin metabolism would result in chronic elevation of the abundance and modified properties of the lipid rafts, a key pathogenic element in many cardiovascular, metabolic and neurological disorders (63–66). Consistent with this idea, monocytes from virologically suppressed HIV-infected individuals were shown to have decreased expression of ABCA1 and reduced cholesterol efflux (67). Given dependence of trained immunity on the duration of the exposure and the concentration of the stimulating agent, it is possible that two different forms of trained immunity may be formed, the first one during initial HIV replication associated with strong inflammatory response, and the second one after commencement of ART, when time of exposure is much longer, but levels of exNef are much lower and only a low-grade inflammation is present. The first form of training may be caused by changes in inflammatory epigenome, while the second – by epigenetic changes affecting lipid rafts. Characterization of these two forms of innate memory training, their mechanisms, interaction and contribution to pathogenesis of different co-morbidities awaits further studies.

## Final considerations

In this Hypothesis and Theory article, we present a new perspective on the pathophysiologic and translational relevance of trained immunity in HIV-associated co-morbidities. To the best of our knowledge, very few, if any, studies have explored this issue. We believe that myeloid cells respond to HIV-1 protein Nef, expressed intracellularly by the infecting virus or delivered by EVs, by becoming primed to increased responses to subsequent activation by inflammatory factors (**Figure 1**). These hyper-responsive cells may be the cause for sustained inflammation, which underlies most co-morbidities associated with HIV infection, even if successfully managed by ART. Future studies will define the molecular details of the underlying mechanisms and may identify therapeutic targets, e.g., lipid rafts, for reversing this effect.

## Data Availability Statement

The original contributions presented in the study are included in the article/supplementary material. Further inquiries can be directed to the corresponding author.

## Author Contributions

DS proposed the idea of several mechanisms of trained immunity initiation, contributed to writing the article and created the figure, YM proposed relationship between the lipid rafts and immunity training, MB initiated and lead writing of the article and finalized the manuscript. All authors contributed to the article and approved the submitted version.

## Funding

The work on this article was supported by the following grants: R01HL140977 and P30AI117970 (MB); R01NS124477 and R01HL158305 (DS and MB), R35HL135737 and R01NS102432 (YM).

## Conflict of Interest

The authors declare that the research was conducted in the absence of any commercial or financial relationships that could be construed as a potential conflict of interest.

## Publisher’s Note

All claims expressed in this article are solely those of the authors and do not necessarily represent those of their affiliated organizations, or those of the publisher, the editors and the reviewers. Any product that may be evaluated in this article, or claim that may be made by its manufacturer, is not guaranteed or endorsed by the publisher.
